# Occurrence of Natural Vertical Transmission of Dengue-2 and Dengue-3 Viruses in *Aedes aegypti* and *Aedes albopictus* in Fortaleza, Ceará, Brazil

**DOI:** 10.1371/journal.pone.0041386

**Published:** 2012-07-25

**Authors:** Victor Emanuel Pessoa Martins, Carlos Henrique Alencar, Michel Tott Kamimura, Fernanda Montenegro de Carvalho Araújo, Salvatore Giovanni De Simone, Rosa Fireman Dutra, Maria Izabel Florindo Guedes

**Affiliations:** 1 Human Biochemistry Laboratory, State University of Ceará, Fortaleza, Ceará, Brazil; 2 Department of Community Health, Federal University of Ceará, Fortaleza, Ceará, Brazil; 3 Center for Genomics and Bioinformatics, State University of Ceará, Fortaleza, Ceará, Brazil; 4 Central Laboratory of Public Health, Ceará State Health Secretariat, Fortaleza, Ceará, Brazil; 5 Peptides and Proteins Laboratory, Instituto Oswaldo Cruz - FIOCRUZ, Rio de Janeiro, Rio de Janeiro, Brazil; 6 Biomedical Engineering Laboratory, Federal University of Pernambuco, Recife, Pernambuco, Brazil; University of Texas Medical Branch, United States of America

## Abstract

**Background:**

*Aedes aegypti* and *Aedes albopictus* perform an important role in the transmission of the dengue virus to human populations, particularly in the tropical and subtropical regions of the world. Despite a lack of understanding in relation to the maintenance of the dengue virus in nature during interepidemic periods, the vertical transmission of the dengue virus in populations of *A. aegypti* and *A. albopictus* appears to be of significance in relation to the urban scenario of Fortaleza.

**Methods:**

From March 2007 to July 2009 collections of larvae and pupae of *Aedes* spp were carried out in 40 neighborhoods of Fortaleza. The collections yielded 3,417 (91%) *A. aegypti* mosquitoes and 336 (9%) *A. albopictus* mosquitoes. Only pools containing females, randomly chosen, were submitted to the following tests indirect immunofluorescence (virus isolation), RT-PCR/nested-PCR and nucleotide sequencing at the C-prM junction of the dengue virus genome.

**Results:**

The tests on pool 34 (35 *A. albopictus* mosquitoes) revealed with presence of DENV-3, pool 35 (50 *A. aegypti* mosquitoes) was found to be infected with DENV-2, while pool 49 (41 *A. albopictus* mosquitoes) revealed the simultaneous presence of DENV-2 and DENV-3. Based on the results obtained, there was a minimum infection rate of 0.5 for A. aegypti and 9.4 for A. albopictus. The fragments of 192 bp and 152 bp related to DENV-3, obtained from pools 34 and 49, was registered in GenBank with the access codes HM130699 and JF261696, respectively.

**Conclusions:**

This study recorded the first natural evidence of the vertical transmission of the dengue virus in populations of *A. aegypti* and *A. albopictus* collected in Fortaleza, Ceará State, Brazil, opening a discuss on the epidemiological significance of this mechanism of viral transmission in the local scenario, particularly with respect to the maintenance of these viruses in nature during interepidemic periods.

## Introduction

Dengue is a mosquito-borne infection that in recent decades has become a major international public health problem, mainly in tropical and subtropical areas [1]. The World Health Organization estimates that 50–100 million people are infected annually with the dengue virus (DENV) worldwide [2]. In Brazil, the first isolated serotype (DENV-1) was in the state of Roraima in 1981; however, in 1986, when this serotype was reintroduced in Brazil, dengue became a major public health problem, and by 2009 more than 5.1 million cases of dengue had been reported [3]. In the same period 389,016 cases of dengue fever were reported in the state of Ceará (northeastern Brazil), of which 42% were recorded in the city of Fortaleza [4]. Between 2007 and 2009, it was identified the circulation of DENV-1, 2 and 3 in Brazil, while in the state of Ceará and Fortaleza city there were identified DENV-2 and 3. In the same period, there were almost 50,000 cases of dengue in Fortaleza and 1.4 million cases in Brazil (Table 1).

**Table 1:** Dengue cases and DENV circulating in Brazil, Ceará and Fortaleza, during 2007 to 2009.

Year	Cases of dengue disease/Dengue virus serotype (DENV)		
	Brazil	Ceará	Fortaleza
2007	475,496/1, 2 and 3	34,359/2 and 3	11,260/2 and 3
2008	585,769/1, 2 and 3	44,244/2 and 3	34,109/2 and 3
2009	393,583/1, 2 and 3	5,144/2	4,210/2

Source: Brazil Health Surveillance Secretariat/Ceará State Health Secretariat/Fortaleza Municipality Health Secretariat.

The dengue virus belongs to the family Flaviviridae, genus Flavivirus, which are phylogenetically related to other important human pathogens, such as the yellow fever (YFV), Japanese encephalitis (JEV), and West Nile (WNV) viruses. The virions are enveloped spherical particles with a single-stranded, positive-sense RNA genome of around 11 kb containing a single open reading frame encoding a single polyprotein co- and post-translationally cleaved into 3 structural (C, prM and E) and 7 nonstructural (NS1, NS2A, NS2B, NS, NS4A, NS4B and NS5) proteins. There are 4 genetically distinct DENV types (DENV-1 to -4), with multiple genotypic variants [5], [6].

Dengue is an arbovirosis transmitted mainly by *Aedes aegypti* and *Aedes albopictus* [7]. *A. aegypti* is a tropical mosquito considered the main vector involved in the urban transmission cycle of the DENV [8]. In Brazil, *A. aegypti* has been responsible for dengue transmission since the early 1980s [9]. *A. albopictus* has adapted to both tropical and temperate climatic regions and has colonized several types of breeding sites in urban and suburban areas [10]. Since it was recorded in Brazil in 1986 [11], *A. albopictus* has not been associated with dengue epidemics in the country, although it has been found naturally infected with YFV and DENV. Laboratory studies have shown their potential to become infected and transmit 20 other arboviruses [12]. In Ceará state, the first record of its presence occurred in 2005 in Fortaleza city [13].

The best known mechanism of DENV transmission is horizontal transmission (human-mosquito); however, transovarial or vertical transmission, where the female-infected mosquito is able to transmit the virus to its progeny, may provide a mechanism to understand how DENV persists in nature in the absence of non-immune vertebrate hosts or under environmental conditions unfavorable for mosquito activity [14], [15].

The current study reports the isolation of DENV-2 and DENV-3 viruses in C6/36 cell cultures of *A. albopictus* and by reverse transcription-polymerase chain reaction (RT-PCR) in pools of *A. aegypti* and *A. albopictus* collected directly from the field in an urban area in Fortaleza city, state of Ceará, Brazil, during the period of 2007 to 2009.

## Methods

### Collection, Maintenance of Immature Forms and Identification of Adult Forms of *Aedes spp*

The city of Fortaleza, capital of Ceará State, is located on the northern coast of the Northeast region of Brazil. With 313.14 km^2^ of surface area and a demographic density of 7,851.27 inhabitants/km^2^, it is currently the fourth largest state capital in terms of population, its total residential population, in 2008, being estimated at 2,458,545 [16]. Since 1997 it has been administratively organized into 6 Regional Executive Secretariats (*Secretarias Executivas Regionais* - SER), in which lie its 116 neighborhoods.

**Figure 1:**
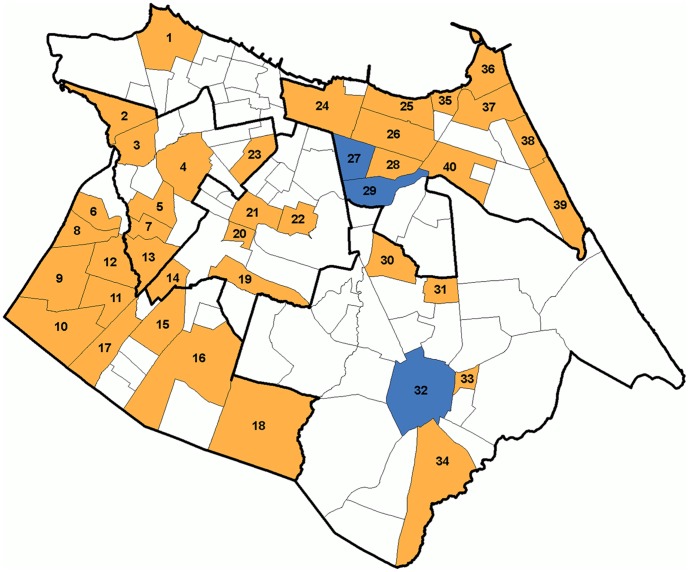
Neighborhoods of Fortaleza where collections of *Aedes spp* were carried out. DENV was isolated from *Aedes* mosquitoes in the neighborhoods colored in blue.

From March 2007 to July 2009, during the activities of the Dengue Control Program in Fortaleza, which are performed every three months, larvae and pupae of *Aedes* spp were collected in households located in 40 neighborhoods of the city (Figure 1). There was no association between the choice of neighborhoods and dengue cases, which were selected according to their infestation rates (Table 2). Parks with a large areas of plant cover frequented by the population for leisure activities were also included in the research. The specimens were separated and transferred to special containers, according to their stage of development. The larvae were kept in plastic containers of 200 mL while the pupae were distributed in 500 mL plastic containers, which were placed inside a cage, adapted for the maintenance of the adult forms. Upon reaching the adult stage mosquitoes were fed only with a sucrose-based solution and, therefore they were completely deprived of having a blood meal. Finally, five days after emergence, adults were identified for the presence of *A. aegypti* and *A. albopictus* [17], while other species of Culicidae were discarded. After identification, mosquitoes were separated into pools of 1 to 50 specimens (according Oswaldo Cruz Foundation protocol’s), by month of collection, and stored at −80°C.

**Table 2:** Infestation rates of neighborhoods of Fortaleza and pools of *A. aegypti* and *A. albopictus* females submitted to IFA and RT-PCR.

Neighborhoods	Infestation rates/Month and year of collection	*A. aegypti* *	*A. albopictus* *
1. Barra do Ceará	3.28/Mar. 2007–1.48/Mar. 2009	50– Mar. 2007	–
2. Quintino Cunha	6.51/Mar. 2007–4.35/Jan. 20083.35/Mar. 2009	50– Mar. 200750– Jan. 2008	48– Mar. 2007
3. Antônio Bezerra	7.27/Mar. 2007–5.74/Jan. 20082.83/June 2009	50– Mar. 2007	-
4. Pici	3.96/Mar. 2007–2.51/Jan 2008	49– Mar. 2007	–
5. Henrique Jorge	5.88/Mar. 2007–3.64/Jan. 20082.54/Mar. 2009	50– Mar. 2009	–
6. Conjunto Ceará I	8.93/Mar. 2007–6.39/Jan. 20084.47/Mar. 2009	50– Jan. 2008	–
7. João XXIII	2.94/Mar. 2007–3.36/June 2009	50– June 2009	–
8. Conjunto Ceará II	6.79/Mar. 2007–5.63/Jan. 20083.98/Mar. 2009	50– Mar. 2007	–
9. Granja Lisboa	4.61/Mar. 2007–5.71/Jan. 2008	50– Jan. 2008	50– Jan. 2008
10. Siqueira	1.33/June 2007–2.34/Mar. 2009	50– June 2007	–
11. Bom Jardim	3.45/Mar. 2007–5.79/Jan. 20082.30/June 2009	49– June 2009	–
12. Granja Portugal	2.95/Mar. 2007–6.76/Jan. 20083.40/June 2009	50– Jan. 2008	–
13. Bom Sucesso	5.08/Mar. 2007–3.86/Jan 20083.45/June 2009	50– Mar. 2007	–
14. Vila Pery	2.65/Mar. 2007–3.24/Jan. 20083.26/Mar. 2009	50– Mar. 2009	–
15. Vila Manoel Sátiro	2.04/Mar. 2007–2.22/Mar. 2009	50– Mar. 2007	–
16. Mondubim	3.12/June 2007–1.76/Mar. 2009	50– June 2007	–
17. Canindezinho	1.88/Mar. 2007–1.46/Mar. 2009	41– Mar. 2007	–
18. José Walter	4.25/Mar. 2007–3.14/Mar. 2009	50– Mar. 2007	–
19. Itaperi	3.24/Mar. 2007–3.51/Jan. 20082.84/Mar. 2009	50– Jan. 2008	–
20. Itaoca	1.34/June 2007–2.16/Jan. 20081.72/Mar. 2009	50– Jan. 2008	–
21. Montese	5.05/Mar. 2007–3.13/Jan. 2008	50– Mar. 2007	18– Mar. 2007
22. Vila União	1.76/Mar. 2007–3.27/Jan. 20082.29/June 2009	50– Jan. 2008	–
23. Rodolfo Teófilo	5.78/Mar. 2007–2.91/Jan. 20084.33/Mar. 2009	50– Mar. 2007	–
24. Centro	3.50/Mar. 2007–4.21/Jan. 20082.67/Mar. 2009	50– Mar. 2009	–
25. Meireles	1.28/Mar. 2007–5.59/Jan. 2008	50– Jan. 2008	–
26. Aldeota	2.89/Mar. 2007–5.97/Jan. 20083.69/Oct. 2008	47– Oct. 2008	–
27. Joaquim Távora	4.78/Mar. 2007–3.34/Jan. 2008	**50– Jan. 2008**	–
28. Dionísio Torres	4.78/Mar. 2007–5.99/Jan. 20082.99/Mar. 2009	47– Mar. 2007	–
29. São João Tauape	3.28/July 2007–2.67/Jan. 2008	24– Jan. 2008	**41– July 2007**
30. Jardim das Oliveiras	1.98/Mar. 2007–1.89/June 2009	48– Mar. 2007	–
31. Parque Manibura	5.32/Dec. 2007–4.11/Jan. 2008	50– Dec. 2007	–
32. Messejana	2.03/May 2007–3.48/Jan. 20082.17/July 2009	50– July 2009	**35– May 2007**
33. Curió	4.00/Mar. 2007–5.59/Jan. 20083.90/June 2009	50– Mar. 2007	–
34. Paupina	2.18/Mar. 2007–1.29/June 2009	50– June 2009	20– June 2009
35. Mucuripe	3.03/Jan. 2008–2.96/Mar. 2009	50– Jan. 2008	–
36. Cais do Porto	2.73/Jan. 2008–1.50/Mar. 2009	50– Jan. 2008	–
37. Vicente Pinzon	3.12/June 2007–2.92/Mar. 2008	50– June 2007	–
38. Praia do Futuro I	5.45/Dec. 2007–1.20/June 2009	50– Dec. 2007	–
39. Praia do Futuro II	1.58/Mar. 2007–2.23/Jan. 2008	50– Jan. 2008	–
40. Cocó	2.23/Jan. 2008	50– Jan. 2008	–

* Mosquitoes/pool – month and year of collection. In bold, positive pools by both IFA and RT-PCR. The other pools were negative for both tests.

### Preparation of Mosquitoes Samples for Virus Isolation and Characterization

Were randomly selected 47 pools of *A. aegypti* and *A. albopictus* females which were macerated in 2 mL microtubes containing 1 mL of L-15 Leibovitz medium (Sigma-Aldrich), supplemented with 100 mL of 2,95% tryptose phosphate (Sigma-Aldrich), 10 mL of a solution of non-essential amino acids (Sigma-Aldrich), 10 mL of 2% L-glutamine (Sigma-Aldrich), and 3 µL of a combined solution of the antibiotics penicillin and streptomycin (10,000 U/mL penicillin G sodium +10,000 µg/µL of streptomycin sulfate in 0.85% saline – Gibco®). Samples were then centrifuged at 2,000×g (30 min at 4°C) and the supernatants were transferred to 1.5 mL microtubes containing 100 mL of penicillin/streptomycin and Amphotericin B (Gibco®) and kept in an ice bath for 2 h. After this period, the samples were centrifuged at 2,000×g (20 min at 4°C). Finally, the supernatants were transferred to new microtubes of 1.5 mL containing 0.3 mL of fetal calf serum (Laborclin®), which were kept at −80°C until virus isolation.

The macerated samples (150 µL) were inoculated into C6/36 cell cultures of *A. albopictus*, according to the protocol established by Igarashi [18]. After two passages (seven days each), cells were subjected to indirect fluorescent antibody test (IFA) using specific monoclonal antibodies for the four dengue virus serotypes, according to the protocol established by Gubler et al. [19]. Cultures of uninfected cells were used as negative controls.

### RNA Extraction, Reverse-transcriptase Polymerase Chain Reaction (RT-PCR) and Nested-PCR

Viral RNA extraction was performed with 250 µL of each cell culture fluids, using the Trizol® LS Reagent (Invitrogen®) method, following the manufacturer’s protocol. RT-PCR for detecting DENV in mosquito pools was performed according to Lanciotti et al. [20], with minor modifications. This technique was used to exclude possible laboratory contamination, and provide additional data for future studies on the degree of variation in the genomic segment used. The first step of RT-PCR consisted of a reverse transcription reaction to synthesize and amplify a 511 bp cDNA fragment from RNA templates, corresponding to the C-prM junction of the dengue virus genome. The consensus primers forward D1 (5′-TCAATATGCTGAAACGCGCGAGAAACCG-3′/Invitrogen®) and reverse D2 (5′-TTGCACCAACAGTCAATGTCTTCAGGTTC-3′/Invitrogen®), and SuperScript™ III (Invitrogen®) and Platinum® Taq DNA polymerase (Invitrogen®) enzymes were used. The second step of the nested-PCR was carried out with D1 and type-specific (TS) reverse primers (TS1: 5′CGTCTCAGTGATCCGGGGG3’; TS2: 5′CGCCACAAGGGCCATGAACAG3’; TS3: 5′TAACATCATCATGAGACAGAGC3’; TS4: 5′ CTCTGTTGTCTTAAACAAGAGA 3′/Invitrogen®), which amplify regions of 482, 119, 290 and 392 bp of DENV-1, DENV-2, DENV-3 and DENV-4, respectively. The detection of amplified fragments was performed by gel electrophoresis (1.5% agarose gel stained with 1% ethidium bromide).

### Estimation of Minimum Infection Rate (MIR)

The minimum infection rate (MIR) of the mosquito pools was calculated from the ratio between the number of positive pools and the total number of mosquitoes tested, multiplied by 1000 [21].

### Nucleotide Sequencing

The PCR products were purified using the Invitrogen PureLink™ kit and sequenced in an ABI Prism 3100 (Applied Biosystems) using a Big Dye Terminator 3.0 kit (California, U.S.) and D1 (forward), TS-2 and TS-3 (reverse) primers, as described by the manufacturer. The nucleic acid sequences were aligned with sequences previously recorded in the GenBank using the Clustal W method of the Megalign Software.

## Results

From the viable samples (live larvae and pupae) obtained from the collections carried out 3,417 (91%) of the specimens belonged to the species *A. aegypti* (1,412 males; 2,005 females) and 336 (9%) belonged to *A. albopictus* (124 males; 212 females).

**Figure 2:**
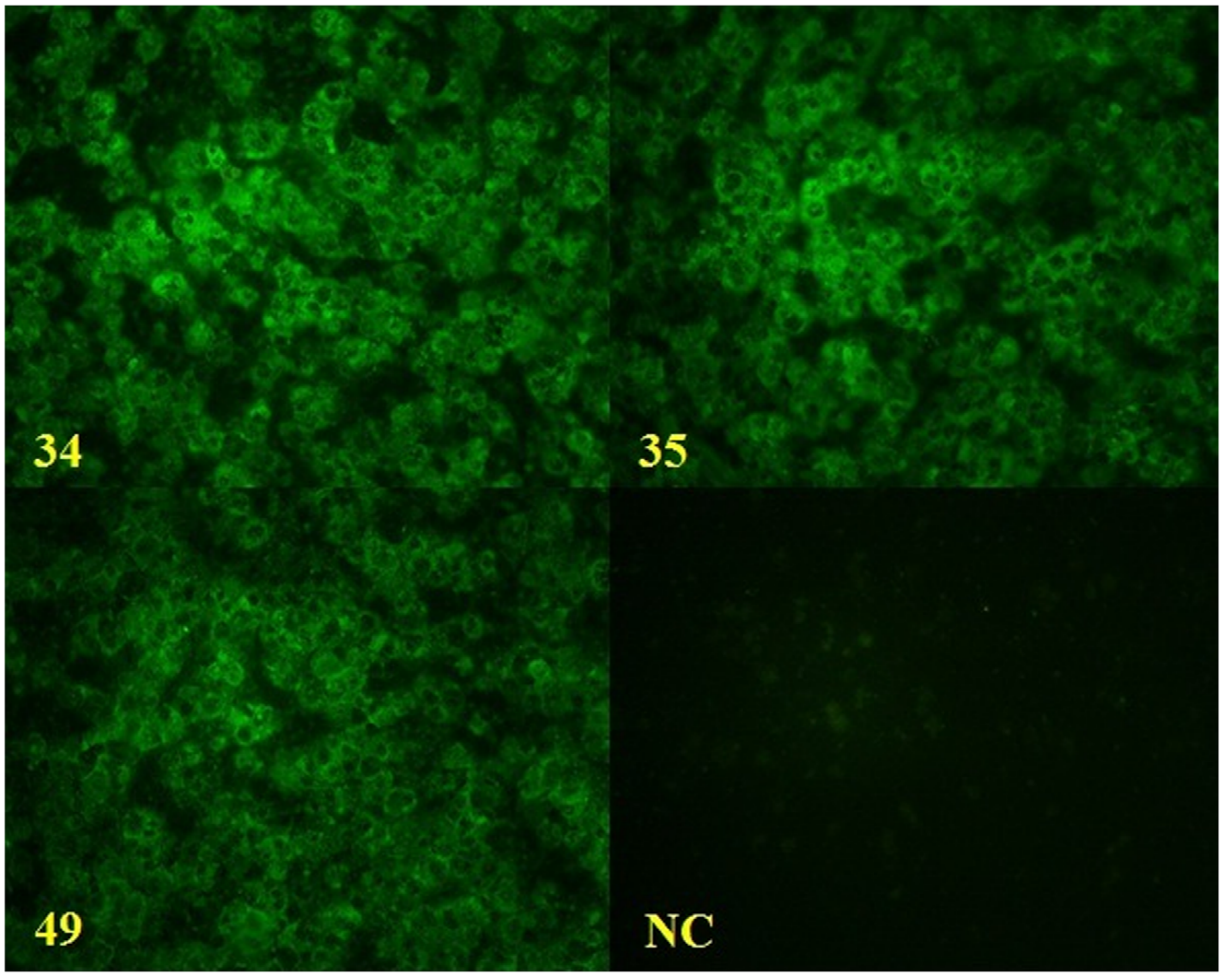
Indirect fluorescent antibody test of *Aedes* mosquitoes pools. Pools 34 (A), 35 (B) and 49 (C) were positive for DENV; NC = negative control.

**Figure 3:**
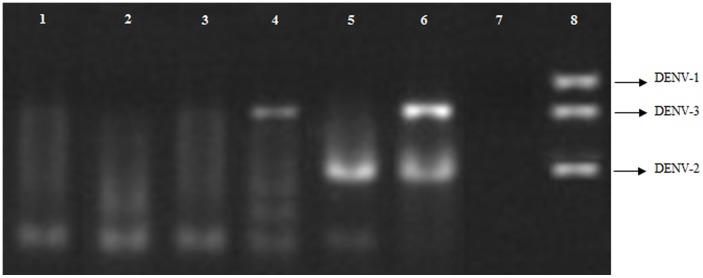
Electrophoresis in agarose gel of the products of RT-PCR. Wells 1, 2 and 3 indicate negative pools for the dengue virus; well 4 reveals genomic fragment of DENV -3, obtained from the pool 34; well 5 reveals genomic fragment of DENV-2 obtained from the pool 35; well 6 reveals simultaneously genomic fragments of DENV-2 and DENV-3, obtained from the pool 49; well 7 contains the negative control, and well 8 contains a mix of DENV-1, DENV-2 and DENV-3 (positive control).

A total of 47 *Aedes* females pools (Table 2) were inoculated in cell cultures, and 3 (6.3%) of them (pools 34, 35 and 49) were positive for DENV by IFA (Figure 2). This result was also confirmed by analysis of the agarose gel electrophoresis profile of these pools submitted to RT-PCR (Figure 3).

Pool 34, comprising 35 *A. albopictus* specimens collected in May 2007 in the neighborhood of Messejana, revealed the presence of DENV-3. Pool 35, comprising 50 *A. aegypti* collected in the neighborhood of Joaquim Távora in January 2008, was found to be infected with DENV-2. Pool 49, comprising 41 *A. albopictus* collected in July 2007 in a park called Parque Adahil Barreto (3° 45′ 16″ S and 38° 30′ 03″ W), located in the neighborhood of São João Tauape, revealed the simultaneous presence of DENV-2 and DENV-3.

From the results obtained, was estimated a minimum infection rate (MIR) of 0.5 (1 positive pool ÷ 2,005 mosquitoes tested × 1000) for *A. aegypti*, while MIR for *A. albopictus* was 9.4 (2 positive pools ÷ 212 mosquitoes tested × 1000).

The nucleotide sequencing of samples relating to DENV-3, obtained from pools 34 and 49, resulted in two fragments of 192 bp and 152 bp, respectively. These sequences were registered in GenBank with the access codes HM130699 and JF261696. The sequence obtained for the DENV-2 showed an electropherogram difficult to be analyzed (overlapping peaks), even after repeated sequencing reactions, thus not included in the results.

## Discussion

The isolation and the genome fragments detection of DENV-2 and DENV-3 in adult females of *A. aegypti* and *A. albopictus*, deprived of having a blood meal, recorded, for the first time, the occurrence of the vertical transmission of DENV in Ceará State.

These facts support the DENV-1 and DENV-2 isolation from *A. albopictus* larvae collected in 2003 in Belo Horizonte, capital of Minas Gerais State (Southeast region of Brazil) [22]. In the same city, DENV-3 was isolated from *A. aegypti* eggs collected during the period of 2000 to 2004 [23]. Guedes et al. [24] isolated DENV-1, DENV-2 and DENV-3 from adult *A. aegypti* females which emerged from eggs collected in Recife, capital of Pernambuco State (Northeast region of Brazil), in 2005 and 2006. Figueiredo et al. [25], in 1999, verified the occurrence of the vertical transmission of DENV-3 in *A. albopictus* larvae, and of DENV-1 in adult females of *A. aegypti*, all specimens originating from the city of Santos, in São Paulo State (Southeast region of Brazil). The same researchers also isolated DENV-2 from female adults of *A. aegypti*, in 2005, in the city of Foz do Iguaçu, in Paraná State (South region of Brazil).

The first report of the circulation of DENV-2 in Ceará State was in 1994, when 47,221 cases of dengue were recorded (26 dengue hemorrhagic fever) [26]. The circulation of this serotype was maintained for the following eight years when, in 2003, DENV-3 was isolated for the first time [27].

Since the appearance of the DENV-3 serotype in Ceará State, the absence of the concomitant circulation of DENV-2 has been observed. This dominance of DENV-3 in relation to DENV-2 has also been observed in Rio de Janeiro State, during the period of 2000–2001, when DENV-3 was detected in 97.8% of the clinical samples submitted to the study [28]. During this study, virological surveillance of the Ceará State detected the circulation of DENV-2 and DENV-3 in Fortaleza by tests performed with sera from human patients [27].

In Brazil, monitoring of DENV by virus isolation and RT-PCR in *Aedes* spp has been performed sporadically by a few researches [29]–[31] and has not been incorporated as a routine activity in dengue control programs. RT-PCR is a powerful tool in virological surveillance of DENV, especially when negative results are obtained by other tests such as virus isolation in cell culture [32]. Based on its high sensitivity, RT-PCR is used in epidemiological studies where large amounts of mosquitoes are collected. Our results corroborate those obtained by Urdaneta et al. [33], in which was observed a high sensitivity level of RT-PCR in *A. aegypti* pool sizes up to 20 *A. aegypti* mosquitoes.

There are different factors that govern the contact between humans and *Aedes* mosquitoes. The exposure rate of humans to the vectors of DENV increases the risk of infection by these viruses [34]. The Adahil Barreto Park has a vast covering of plants in its surrounds, enabling favorable conditions for the appearance and maintenance of *A. albopictus*, as observed by Alencar [unpublished data]. This area shows an intense flux of people, serving as a place for the practicing of sports and other leisure activities by the population, it is an excellent point of contact between humans and specimens of *A. albopictus* which can take advantage of this situation to carry out their blood sucking.

Thus, the detection of DENV in specimens of *A. albopictus* prompts discussion regarding the transmission of DENV to the people who visit the Adahil Barreto Park and its dissemination to other areas, mainly when they return to their homes, where there is a predominance of *A. aegypti* [35]. This potential of *A. albopictus* to act as a bridge for the introduction of this arbovirus in peridomestic environments is a factor which increases the risk of human infection [36].

In Brazil, there have been reports of the isolation of DENV from populations of *A. aegypti* in Distrito Federal (Center-West region of Brazil) [37], in Nova Iguaçu (Rio de Janeiro State – Southeast region of Brazil) [30], and in Manaus (capital of Amazonas State – North region of Brazil) [29], [38]. However, these findings did not document the phenomenon of the vertical transmission of DENV in *A. aegypti* occurring in Brazil, since the females submitted to the virus isolation tests had not been deprived of blood feeding. On the other hand, in our study, the detection of DENV in adult females deprived of blood feeding reinforces the vertical (transovarian) transmission of DENV in *Aedes* mosquitoes as an important mechanism in the maintenance of this virus in nature.

There have been several reports on the vectorial competence and capacity of different populations of *A. aegypti* and *A. albopictus* in relation to DENV. In many of these studies the mosquitoes were infected artificially with different strains and serotypes of DENV and, in parallel, attempts were made to observe the occurrence of the vertical transmission of this virus to progeny [15], [39]–[48]. On the other hand, there are some references regarding to the natural occurrence of the vertical transmission of DENV in the species *A. aegypti* and *A. albopictus.* In these studies, it was possible to identify DENV in adult mosquitoes emerging from both larvae and eggs collected in the field, deprived of a blood feeding, and adult collected directly in the field (Table 3) [14], [49]–[64].

**Table 3:** Records of the occurrence of vertical transmission of DENV in *A. aegypti* and *A. albopictus*.

Stage of insect development	Species	DENV isolate/Detected by	Country/Reference
aAdult (♂)	*A. aegypti*	2/head squash-direct fluorescence antibody	Myanmar (14)
bAdult *	*A. aegypti*	4/indirect fluorescence antibody and complement fixation	Trinidad and Tobago (49)
aAdult *	*A. aegypti*	3/indirect fluorescence antibody	India (50)
cAdult (♂)	*A. albopictus*	2, 3/indirect fluorescence antibody and RT-PCR	Mexico (51)
cAdult (♂ and ♀)	*A. aegypti* and *A. albopictus*	1/RT-PCR	Singapore (52)
aAdult (♂ and ♀)	*A. aegypti*	2, 3/ELISA and *Toxorhynchites splendens*inoculation-indirect immunofluorescence	India (53)
Larvae	*A. aegypti* and *A. albopictus*	♦/peroxidase anti peroxidase staining	Malaysia (54)
cAdult (♂)	*A. aegypti* and *A. albopictus*	1, 2, 3, 4/RT-PCR	Singapore (55)
aAdult (♀)	*A. aegypti*	2, 3, 4/RT-PCR	Mexico (56)
Larvae and aadult (♂ and ♀)	*A. albopictus*	2/ELISA and indirect fluorescence antibody	India (57)
Larvae	*A. aegypti* and *A. albopictus*	1, 3/Indirect fluorescence antibody and RT-PCR	Malaysia (58)
Larvae and aadult (♂ and ♀)	*A. aegypti*	2/RT-PCR	Indonesia (59)
aAdult *	*A. aegypti* and *A. albopictus*	♦/Indirect fluorescence antibody	India (60)
cAdult (♂)	*A. aegypti*	2, 3/ELISA and *Toxorhynchites splendens*inoculation-indirect immunofluorescence	India (61)
aAdult *	*A. aegypti*	♦/head squash- direct fluorescence antibody	Indonesia (62)
aAdult (♂ and ♀)	*A. aegypti* and *A. albopictus*	♦/ELISA	India (63)
aAdult *	*A. aegypti*	1,2, 3, 4/RT-PCR	Thailand (64)

a Adults that emerged from larvae collected in the field;

b Adults that emerged from eggs collected in the field;

c Adults collected in the field;

(*)No information available on the sex of the mosquitoes tested;

(♦)Serotypes not specified.

The epidemiological relevance of the role of vector mosquitoes in the transmission of this arbovirus within a certain period can be estimated through the minimum infection rate (MIR), which may serve as a tool for predicting epidemics [65]. The MIR values observed in our study points to its useful for the prediction of epidemic episodes of dengue, since in 2008 the largest epidemic of the disease in Fortaleza was recorded, with the circulation of DENV-2 and DENV-3, and 34,109 confirmed cases [4]. This is a notable result since *A. albopictus* is not considered as a vector of the dengue virus in Brazil. Surveillance of DENV vectors allows timely implementation of emergency mosquito control measures such as insecticidal fogging of adults and destruction of breeding sites to inhibit an impending outbreak from spreading [52].

Considering these results and the adaptive potential of both species to colonize a wide variety of types of breeding sites in the urban environment of Fortaleza, as demonstrated by Martins et al. [35], it is necessary to expand the strategies directed toward combating these Culicidae in the Dengue Control Programs in Fortaleza, especially in relation to *A. albopictus*. Although there are no confirmed cases in the literature of the transmission of DENV by populations of *A. albopictus* in episodes of epidemics in Brazil, this possibility cannot be discarded. Furthermore, the occurrence of the vertical transmission of DENV-2 and DENV-3 in *A. aegypti* and *A. albopictus* in Fortaleza opens discussion regarding the role performed by this viral transmission mechanism in the maintenance of DENV in nature during interepidemic periods in Brazil.

## References

[1] Kyle JL, Harris E (2008). Global spread and persistence of dengue.. Annu Rev Microbiol.

[2] World Health Organization (2009). Dengue and Dengue haemorrhagic fever.. Fact sheet No. 117, Geneva.

[3] Teixeira MG, Costa MCN, Barreto F, Barreto ML (2009). Dengue: vinte e cinco anos da reemergência no Brasil. Cad.. Saúde Pública, Rio de Janeiro, 25 Sup.

[4] Fortaleza Municipality Health (2009). Plano de Contingência para o Controle da Dengue no Município de Fortaleza. Available: http//sms.fortaleza.ce.gov.br/sms.. Accessed 11 Oct 2010.

[5] Chambers TJ, Hahn C, Galler R, Rice CM (1990). Flavivirus genome organization, expression and replication.. Annu Rev Microbiol.

[6] Rico-Hesse R (2003). Microevolution and virulence of dengue viruses.. Adv Virus Res.

[7] Rodhain F, Rosen L, Gubler DJ, Kuno G (1997). Mosquito vectors and dengue virus-vector relationships..

[8] Gubler DJ (1998). Dengue and dengue hemorrhagic fever.. Clin Microbiol Rev.

[9] Lourenco-de-Oliveira R, Vazeille M, de Filippis AM, Failloux AB (2004). Aedes aegypti in Brazil: genetically differentiated populations with high susceptibility to dengue and yellow fever viruses.. Trans R Soc Trop Med Hyg.

[10] Hawley WA (1988). The biology of *Aedes albopictus.*. J Am Mosq Control Assoc.

[11] Forattini OP (1986). Identification of *Aedes (Stegomyia) albopictus* (Skuse) in Brazil.. Rev Saúde Pública.

[12] Moore CG, Mitchell CJ (1997). *Aedes albopictus* in the United States: ten-year presence and public health implications.. Emerg Infect Dis.

[13] Martins VEP, Martins MG, Araújo JMP, Silva LOR, Monteiro HAO (2006). Primeiro registro de *Aedes (Stegomyia) albopictus* no Estado do Ceará, Brasil.. Rev Saúde Pública.

[14] Khin MM, Than KA (1983). Transovarial transmission of dengue 2 virus by *Aedes aegypti* in nature.. Am J Trop Med Hyg.

[15] Rosen L, Shroyer DA, Tesh RB, Freier JE, Lien JC (1983). Transovarial transmission of dengue virus by mosquitoes: *Aedes albopictus* and *Aedes aegypti*.. Am J Trop Med Hyg.

[16] (2009). Instituto Brasileiro de Geografia e Estatística.. Accessed 15 Jan.

[17] Consoli R, Lourenço-de-Oliveira R (1994). Principais mosquitos de importância sanitária no Brasil. Rio de Janeiro: Fundação Instituto Oswaldo Cruz.. 225 p.

[18] Igarashi A (1978). Isolation of a Singh’s *Aedes albopictus* cell clone sensitive to dengue and chikungunya viruses.. J Gen Virol.

[19] Gubler DJ, Kuno G, Sather GE, Velez M, Oliver A (1984). Use of mosquito cell cultures and specific monoclonal antibodies in surveillance for dengue viruses.. Am J Trop Med Hyg.

[20] Lanciotti RS, Calisher CH, Gubler DJ, Chang G, Vorndam V (1992). Rapid detection and typing of dengue viruses from clinical samples by using reverse transcriptase-polymerase chain reaction.. J Clin Microbiol.

[21] Savage HM, Smith GC, Moore CG, Mitchell CJ, Townsend M (1993). Entomologic investigations of an epidemic of St. Louis encephalitis in Pine Bluff, Arkansas, 1991.. J Am Mosq Control Assoc.

[22] Cecílio AB, Campanelli ES, Souza KP, Figueiredo LB, Resende MC (2009). Natural vertical transmission by *Stegomyia albopicta* as dengue vector in Brazil.. Braz J Biol 123–127.

[23] Vilela APP, Figueiredo LB, Santos JR, Eiras AE, Bonjardim CA (2010). Dengue virus 3 genotype I in *Aedes aegypti* mosquitoes and eggs, Brazil, 2005–2006.. Emerg Infect Dis.

[24] Guedes DRD, Cordeiro MT, Melo-Santos MAV, Magalhães T, Marques E (2010). Patient-based dengue surveillance in *Aedes aegypti* from Recife, Brazil.. J Vector Borne Dis.

[25] Figueiredo MLG, Gomes AC, Amarilla AA, Leandro AS, Orrico AS (2010). Mosquitoes infected with dengue viruses in Brazil.. Virol J.

[26] Vasconcelos PFC, Menezes DB, Melo LP, Pessoa ETF, Rodrigues SG (1995). A large epidemic of dengue fever with dengue hemorrhagic cases in Ceará State, Brazil, 1994.. Rev Inst Med Trop São Paulo.

[27] (2010). Ceará State Health Secretariat. Informe Semanal do Dengue. Fortaleza, 27/08/2010.. Accessed 29 Aug.

[28] Nogueira RMR, Araújo JM, Schatzmayr HG (2007). Dengue viruses in Brazil, 1986–2006.. Rev Panam Salud Pública.

[29] Pinheiro VCS, Tadei WP, Barros PMSS, Vasconcelos PFC, Cruz ACR (2005). Detection of dengue virus serotype 3 by reverse transcription-polymerase chain reaction in *Aedes aegypti* (Diptera: Culicidae) captured in Manaus, Amazonas.. Mem Inst Oswaldo Cruz.

[30] Lourenço-de-Oliveira R, Honório NA, Castro MG, Schatzmayr HG, Miagostovich MP (2002). Dengue virus type 3 isolation from *Aedes aegypti* in the municipality of Nova Iguaçu, state of Rio de Janeiro.. Mem Inst Oswaldo Cruz.

[31] Zeidler JD, Acosta POA, Barrêto PP, Cordeiro JS (2008). Vírus dengue em larvas de *Aedes aegypti* e sua dinâmica de infestação, Roraima, Brasil.. Rev Saúde Pública.

[32] Miagostovich MP, Santos FB, Araújo ESM, Dias J, Schatzmayr HG (1997). Diagnosis of dengue by using reverse transcriptase-polimerase chain reaction.. Mem Inst Oswaldo Cruz.

[33] Urdaneta L, Herrera F, Pernalete M, Zoghbi N, Rubio-Palis Y (2005). Detection of dengue viroses in field-caught Aedes aegypti (Diptera: Culicidae) in Maracay, Aragua state, Venezuela by type-specific polimerase chain reaction.. Infec Genet Evol.

[34] Stoddard ST, Morrison AC, Vazquez-Prokopec GM, Paz Soldan V, Kochel TJ (2009). The Role of Human Movement in the Transmission of Vector-Borne Pathogens.. PLoS Negl Trop Dis.

[35] Martins VEP, Alencar CHM, Facó PEG, Dutra RF, Alves CR (2010). Distribuição espacial e características dos criadouros de *Aedes albopictus* e *Aedes aegypti* em Fortaleza, Estado do Ceará.. Rev Soc Bras Med Trop.

[36] Lambrechts L, Scott TW, Gubler DJ (2010). Consequences of the expanding global distribution of *Aedes albopictus* for dengue virus transmission.. PLoS Negl Trop Dis.

[37] Degallier N, Teixeira JMS, Vilarinhos PTR, Pinto SCF, Pereira RD (2000). First isolation of dengue 1 virus from *Aedes aegypti* in Federal District, Brazil.. Rev Soc Bras Med Trop.

[38] Costa CA, Santos IGC, Barbosa MG (2009). Detecção e tipagem de vírus dengue em *Aedes aegypti* (Diptera: Culicidae) na Cidade de Manaus, Estado do Amazonas.. Rev Soc Bras Med Trop.

[39] Jousset FX (1981). Geographic *Aedes aegypti* strains and dengue-2 virus: susceptibility, ability to transmit to vertebrate and transovarial transmission.. Annales de l’Institut Pasteur.

[40] Shroyer DA (1990). Vertical maintenance of dengue-1 virus in sequential generations of *Aedes albopictus*.. J Am Mosq Control Assoc.

[41] Rosen L (1988). Further observations on the mechanism of vertical transmission of flaviviruses by Aedes mosquitoes.. Am J Trop Med Hyg.

[42] Mitchell CJ, Miller BR (1990). Vertical transmission of dengue viroses by strains of *Aedes albopictus* recently introduced into Brazil.. J Am Mosq Control Assoc.

[43] Bosio CF, Thomas RE, Grimstad PR, Rai KS (1992). Variation in the efficiency of vertical transmission of dengue-1 virus by strains of *Aedes albopictus* (Diptera: Culicidae).. J Med Entomol.

[44] Mourya DT, Gokhale MD, Basu A, Barde PV, Sapkal GN (2001). Horizontal and vertical transmission of dengue virus type 2 in highly and lowly susceptible strains of Aedes aegypti mosquitoes.. Acta Virol.

[45] Joshi V, Mourya DT, Sharma RC (2002). Persistence of dengue-3 virus through transovarial transmission passage in successive generations of *Aedes aegypti* mosquitoes.. Am J Trop Med Hyg.

[46] Wasinpiyamongkol L, Thongrungkiat S, Jirakanjanakit N, Apiwathnasorn C (2003). Susceptibility and transovarial transmission of dengue virus in *Aedes aegypti*: a preliminar study of morphological variations.. Southeast Asian J Trop Med Public Health.

[47] Castro MG, Nogueira RMR, Schatzmayr HG, Miagostovich MP, Lourenço-de-Oliveira R (2004). Dengue virus detection by using reverse transcription –polymerase chain reactions in saliva and progeny of experimentally infected *Aedes albopictus* from Brazil.. Mem Inst Oswaldo Cruz.

[48] Rohani A, Zamree I, Joseph RT, Lee HL (2008). Persistency of transovarial dengue virus in *Aedes aegypti* (Linn).. Southeast Asian J Trop Med Public Health.

[49] Hull B, Tikasingh E, Souza M, Martinez R (1984). Natural transovarial transmission of dengue 4 virus in *Aedes aegypti* in Trinidad.. Am J Trop Med Hyg.

[50] Joshi V, Singhi M, Chaudhary RC (1996). Transovarial transmission of dengue 3 virus by *Aedes aegypti*.. Trans R Soc Trop Med Hyg.

[51] Ibañez-Bernal S, Briseño B, Mutebi JP, Argot E, Rodriguez G (1997). First Record in America of *Aedes albopictus* naturally infected with dengue virus during 1995 outbreak at Reynosa, Mexico.. Med Vet Entomol.

[52] Chow VT, Chan YC, Yong R, Lee KM, Lim LK (1998). Monitoring of dengue viruses in field-caught Aedes aegypti and Aedes albopictus mosquitoes by a type-specific polymerase chain reaction and cycle sequencing.. Am J Trop Med Hyg.

[53] Thenmozhi V, Tewari SC, Manavalan R, Balasubramanian A, Gajanana A (2000). Natural vertical transmission of dengue viruses in *Aedes aegypti* in southern India.. Trans R Soc Trop Med Hyg.

[54] Lee HL, Rohani A (2005). Transovarial transmission of dengue virus in *Aedes aegypti* and *Aedes albopictus* in relation to dengue outbreak in an urban area in Malaysia.. Dengue Bulletin.

[55] Kow CY, Koon LL, Yin PF (2001). Detection of dengue viruses in field-caught male *Aedes aegypti* and *Aedes albopictus* (Diptera: Culicidae) in Singapore by type-specific PCR.. J Med Entomol.

[56] Gunther J, Martínez-Muñoz JP, Pérez-Ishiwara DG, Salas-Benito J (2007). Evidence of vertical transmission of dengue virus in two endemic localities in the state of Oaxaca, Mexico.. Intervirology.

[57] Thenmozhi V, Hiriyan JG, Tewari SC, Samuel PP, Paramasivan R (2007). Natural vertical transmission of dengue virus in *Aedes albopictus* (Diptera: Culicidae) in Kerala, a southern Indian state.. Jpn J Infect Dis.

[58] Rohani A, Zamree I, Lee HL, Mustafakamal I, Norjaiza MJ (2007). Detection of transovarial dengue virus from field-caught *Aedes aegypti* and *Aedes albopictus* larvae using C6/36 cell culture and reverse transcriptase-polymerase chain reaction (RT-PCR) techniques.. Dengue Bulletin.

[59] Akbar MR, Agoes R, Djatie T, Kodyat S (2008). PCR detection of dengue transovarial transmissibility in *Aedes aegypti* in Brandung, Indonesia.. Proc ASEAN Congr Trop Med Parasitol.

[60] Angel B, Joshi V (2008). Distribution and seasonality of vertically transmitted dengue viruses in *Aedes* mosquitoes in arid and semi-arid areas of Rajasthan, India.. J Vector Borne Dis.

[61] Arunachalam N, Tewari SC, Thenmozhi V, Rajedran R, Paramasivan R (2008). Natural vertical transmission of dengue viruses by *Aedes aegypti* in Chennai, Tamil Nadu, India.. Indian J Med Res.

[62] Mardihusodo SJ, Satoto TBT, Mulyaningsih B (2008). Transovarial transmission of dengue virus in *Aedes aegypti* (Diptera: Culicidae) in Yogyakarta, Indonesia.. Epidemiol Mol Evol 297.

[63] Kumari R, Kumar K, Chauhan LS (2011). First dengue virus detection in *Aedes albopictus* from Delhi, India: Its breeding ecology and role in dengue transmission.. Trop Med Int Health 1–6.

[64] Thongrungkiat S, Maneekan P, Wasinpiyamongkol L, Prummongkol S (2011). Prospective field study of transovarial dengue-virus transmission by two different forms of *Aedes aegypti* in an urban area of Bangkok, Thailand.. J Vect Ecol.

[65] Boromisa RD, Grayson MA (1990). Incrimination of *Aedes provocans* as a vector of Jamestown Canyon virus in an enzootic focus of northeastern New York.. J Am Mosq Control Assoc.

