# Correction: Occurrence of Natural Vertical Transmission of Dengue-2 and Dengue-3 Viruses in *Aedes aegypti* and *Aedes albopictus* in Fortaleza, Ceará, Brazil

**DOI:** 10.1371/annotation/bc186d1e-f2fc-4dff-8084-a25cf32b9388

**Published:** 2012-08-23

**Authors:** Victor Emanuel Pessoa Martins, Carlos Henrique Alencar, Michel Toth Kamimura, Fernanda Montenegro de Carvalho Araújo, Salvatore Giovanni De Simone, Rosa Fireman Dutra, Maria Izabel Florindo Guedes

There was an error in the third author's name. Michel Toth Kamimura is correct. 

